# The immunoregulatory effects of CMV-infection in human fibroblasts and the impact on cellular senescence

**DOI:** 10.1186/1742-4933-9-1

**Published:** 2012-03-29

**Authors:** Juliane Wolf, Birgit Weinberger, Beatrix Grubeck-Loebenstein

**Affiliations:** 1 Immunology Division, Institute for Biomedical Aging Research, Austrian Academy of Sciences, Rennweg 10, 6020 Innsbruck, Austria

**Keywords:** Cytomegalovirus, Aging, Fibroblasts, Replicative senescence

## Abstract

**Background:**

As a chronic antigenic stressor human Cytomegalovirus (CMV) contributes substantially to age-related alterations of the immune system. Even though monocytes have the greatest propensity for CMV-infection and seem to be an important host for the virus during latency, fibroblasts are also discussed to be target cells of CMV *in vivo*. However, little is known so far about general immunoregulatory properties of CMV in fibroblasts. We therefore investigated the immunoregulatory effects of CMV-infection in human lung fibroblasts and the impact on replicative senescence.

**Findings:**

We observed that CMV-infection led to the induction of several immunoregulatory host cell genes associated with the innate and adaptive immune system. These were genes of different function such as genes regulating apoptosis, cytokines/chemokines and genes that are responsible for the detection of pathogens. Some of the genes upregulated following CMV-infection are also upregulated during cellular senescence, indicating that CMV causes an immunological phenotype in fibroblasts, which is partially reminiscent of replicative senescent cells.

**Conclusion:**

In summary our results demonstrate that CMV not only affects the T cell pool but also induces inflammatory processes in human fibroblasts.

## Introduction

Cytomegalovirus (CMV) is a ubiquitous beta-herpesvirus with a worldwide prevalence of 60-100% in the adult population [1]. Infection occurs early and leads to life-long persistence in the host. CMV is one of the most immunodominant antigens and stimulates immune responses of unprecedented magnitude [2]. Several studies have shown that latent infection with cytomegalovirus contributes to age-related alterations of the immune system, particularly of the T cell compartment as it drives the differentiation of T cells and accelerates immunosenescence [3]. In the human host CMV exhibits tropism among others for monocytes/macrophages, fibroblasts and endothelial cells [4-6]. Previous reports demonstrate that CMV induces premature senescence in early passage human fibroblasts. Similar to senescent cells, which have reached the limit of their replicative capacity [7], CMV-infected fibroblasts show intense senescence-associated ß-Galactosidase (SA-ß-gal) activity and increased mRNA expression of the cell cycle arrest gene p16 [8,9]. Replicatively senescent fibroblasts characteristically also produce increased levels of inflammatory molecules [10]. They may thus contribute to the development of subclinical age-related inflammatory processes ('inflamm-aging') [11] and are believed to support the development of age-related diseases [12]. It is an interesting and to our knowledge not yet addressed question, whether CMV-infection of human fibroblasts not only triggers replicative senescence, but also induces the inflammatory phenotype characteristic for this differentiation stage. This may be a trigger for a dysbalance between pro- and antiinflammatory mechanisms and accelerate immunosenescence from early life onwards. Therefore the aim of this study was to investigate the effect of CMV on the expression of genes associated with innate and adaptive immune response in human fibroblasts and to analyze if the expression of these genes causes an inflammatory state which is equal to that of replicative senescent cells.

## Results and discussion

Previous work with fibroblasts showed that cellular senescence is associated with changes in gene expression, particularly of the cellular secretome (senescence-associated secretory phenotype; SASP) [10,13,14]. In our study a broad analysis of the mRNA expression of immunity-related genes in human lung fibroblasts of different passages of cultivation was carried out using the RT^2 ^Profiler PCR Array. We observed that 28 genes out of 84 investigated genes were differentially expressed in early versus late passage fibroblasts (see Table 1) supporting previous results of senescence-associated changes in gene expression. These are genes of different functional groups, with mainly genes for the detection of pathogens (e.g. TLR4), cytokines (e.g. IL6) or the innate immune response (e.g. PGLYRP3) being upregulated.

**Table 1:** Differently expressed genes in early versus replicative senescent and CMV-infected versus untreated human lung fibroblasts

	Gene symbol	Gene name	Fold regulation in replicative senescent fibroblasts (mean ± S.E.M.)	Maximally observed fold regulation (mean ± S.E.M.) following CMV-infection
**Apoptosis**	CASP1	Caspase 1	-	2.4 ± 1.3
	
	CASP4	Caspase 4	-	2.3 ± 1.3
	
	TGFB1	Transforming growth factor, beta 1	-	2.1 ± 0.9
	
	TNFRSF1A	Tumor necrosis factor receptor superfamily, member 1A	-	2.4 ± 1.6

**Complement activation**	C5	Complement component 5	-	2.3 ± 1.5
	
	C8A	Complement component 8, alpha polypeptide	6.8 ± 0.8	-
	
	CD55	CD55 molecule, accelerating factor for complement	-	4.3 ± 0.9

**Cytokines, chemokines and their receptors**	CCL2	Chemokine (C-C motif) ligand 2	-	2.8 ± 1.8
	
	CXCR4	Chemokine receptor 4	-	57.8 ± 1.5
	
	IFNA1	Interferon, alpha 1	3.7 ± 0.3	-
	
	**IFNGR1**	**Interferon gamma receptor 1**	2.0 ± 0.4	3.2 ± 1.6
	
	IFNGR2	Interferon gamma receptor 2	-	2.2 ± 1.6
	
	**IL1A**	**Interleukin 1, alpha**	6.4 ± 0.2	6.4 ± 1.6
	
	**IL1B**	**Interleukin 1, beta**	4.1 ± 0.1	8.9 ± 1.7
	
	IL1F5	Interleukin 1 family, member 5	3.2 ± 1.2	-
	
	IL1F7	Interleukin 1 family, member 7	8.9 ± 0.8	-
	
	**IL6**	**Interleukin 6 (interferon, beta 2)**	10.2 ± 0.04	8.1 ± 1.5
	
	TNF	Tumor necrosis factor	3.2 ± 0.4	-

**Detection of pathogens**	TLR2	Toll-like receptor 2	-	6.1 ± 5.4
	
	TLR3	Toll-like receptor 3	-	5.9 ± 3.9
	
	**TLR4**	**Toll-like receptor 4**	3.8 ± 0.9	5.4 ± 4.7
	
	TLR6	Toll-like receptor 6	3.0 ± 0.2	-
	
	TOLLIP	Toll interacting protein	-	2.5 ± 1.3

**Defense response**	CAMP	Cathelicidin antimicrobial peptide	8.3 ± 0.6	-
	
	FN1	Fibronectin 1	2.5 ± 0.2	-

**IL1receptor pathway**	IL1R1	Interleukin 1 receptor, type I	-	2.9 ± 1.2
	
	IL1RL2	Interleukin 1 receptor-like 2	-	5.3 ± 2.5
	
	IL1RAP	Interleukin 1 receptor accessory protein	-	2.4 ± 1.8
	
	IKBKB	Inhibitor of kappa light polypeptide gene enhancer	2.8 ± 0.2	-
	
	MAPK8	Mitogen activated protein kinase 8	-	2.5 ± 1.5
	
	MAPK14	Mitogen activated protein kinase 14	-	2.5 ± 1.3

**Inflammatory response**	**ADORA2A**	**Adenosine A2a receptor**	12.2 ± 3.0	4.2 ± 4.2
	
	CCR3	Chemokine (C-C motif) receptor 3	12.3 ± 0.8	-
	
	CD14	CD14 molecule	29.1 ± 1.0	-
	
	CYBB	Cytochrome b-245, beta polypeptide	8.5 ± 0.5	-
	
	IRAK1	Interleukin-1 receptor-associated kinase 1	-	3.2 ± 1.7
	
	**IRAK2**	**Interleukin-1 receptor-associated kinase 2**	2.4 ± 0.04	2.7 ± 1.5
	
	NOS2A	Nitric oxide synthase 2A (inducible)	4.7 ± 0.9	-
	
	PTAFR	Platelet-activating factor receptor	4.5 ± 1.4	-

**Innate immune response**	DMBT1	Deleted in malignant brain tumors 1	8.9 ± 1.4	-
	
	PGLYRP1	Peptidoglycan recognition protein 1	10.7 ± 1.8	-
	
	PGLYRP3	Peptidoglycan recognition protein 3	12.0 ± 1.1	-
	
	SFTPD	Surfactant, pulmonary-associated protein D	10.0 ± 1.1	-

**NFkB pathway**	CHUK	Conserved helix-loop-helix ubiquitous kinase	-	2.1 ± 1.3
	
	MyD88	Myeloid differentiation primary response gene	-	3.9 ± 1.8
	
	NFKB2	Nuclear factor of kappa light polypeptide gene enhancer in B cells 2 (p49/p100)	-	3.2 ± 1.6
	
	NFKBIA	Nuclear factor of kappa light polypeptide gene enhancer in B cells inhibitor, alpha	-	2.0 ± 0.9
	
	TRAF6	TNF receptor-associated factor 6	-	2.3 ± 1.5

**Other group**	COLEC12	Collectin subfamily member 12	2.2 ± 0.2	-
	
	HMOX1	Heme oxygenase 1	-	2.0 ± 1.0
	
	IRF1	Interferon regulatory factor 1	-	3.4 ± 1.8
	
	LY96	Lymphocyte antigen 96	-	2.6 ± 1.7
	
	**NLRC4**	**NLR family, CARD domain containing 4**	2.3 ± 0.2	2.7 ± 1.6
	
	**TREM1**	**Triggering receptor expressed on myeloid cells 1**	4.1 ± 1.1	10.0 ± 2.8

The fold regulation data represent the mean ± S.E.M. of three independent experiments calculated for each examined group and time point.

**Figure 1:**
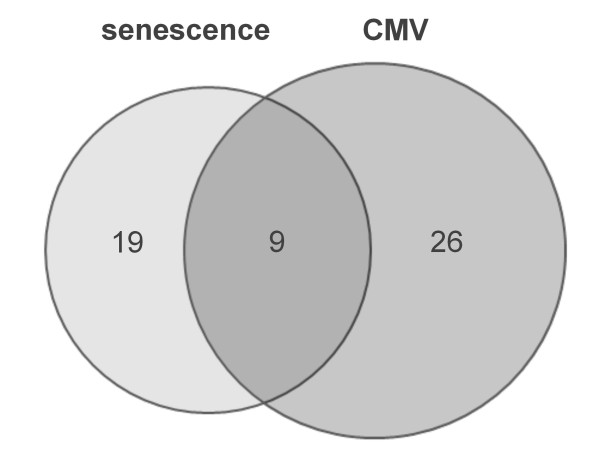
**BioVenn diagram showing the number of immunity-related genes upregulated in replicative senescent and CMV-infected fibroblasts**. Shown is the number of genes differentially expressed in late passage compared to early passage fibroblasts (senescence) and the number of genes differentially expressed in CMV-infected versus untreated fibroblasts (CMV). 9 genes are regulated in both analyses. Depicted are genes that were reproducibly regulated in three independent experiments.

**Figure 2:**
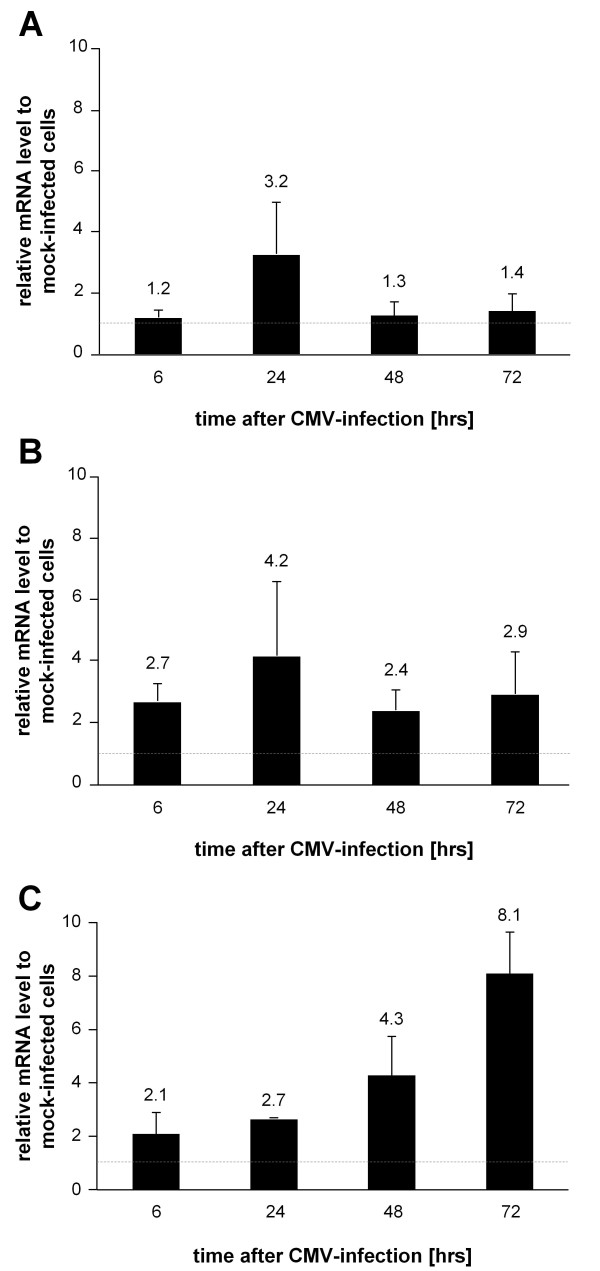
**mRNA levels of IFNGR1, ADORA2A and IL6 over time in CMV-infected fibroblasts**. Human lung fibroblasts were *in vitro *infected with CMV. Cells were harvested at the indicated time points, total RNA was isolated and gene expression of (A) IFNγ receptor 1 (IFNGR1), (B) adenosine A2A receptor (ADORA2A) and (C) Interleukin-6 (IL6) was analyzed using a RT^2 ^Profiler PCR Array. Gene expression of CMV-infected fibroblasts was calculated in relation to gene expression of mock-infected fibroblasts. IFNGR1 and ADORA2A were maximally upregulated 24 hours post infection. While IFNGR1 expression returned to basal levels after 48 hours, ADORA2A was relatively stable expressed over time. In contrast IL6 showed a continuous upregulation of gene expression with a maximum at 72 hours following CMV-infection. The arithmetic mean ± S.E.M. were calculated for each examined group and time point (three independent experiments).

Previous studies showed that CMV induces premature senescence of infected cells [8,9]. In order to elucidate whether CMV also influences the immunological phenotype, the expression of a panel of genes in CMV-infected fibroblasts using a RT^2 ^Profiler PCR Array was analyzed. We could show that *in vitro *CMV-infection of early passage fibroblasts leads to the induction of 35 inflammatory genes. 9 of these genes ( = 25.7%, see Figure 1) upregulated following CMV-infection are genes that are also upregulated during replicative senescence of human fibroblasts (Table 1 depicted in bold), indicating that CMV causes an immunological phenotype in early passage fibroblasts which is to some extent equal to that of uninfected replicative senescent cells. The probability that this simultaneous regulation of genes associated in senescence and CMV-infection was just chance was below 10% and could have been even lower if more genes and not only immunoregulatory ones had been investigated. Most similarities were found in the regulation of genes coding for cytokines, chemokines and their receptors with the upregulation of IL6 being the most prominent example. In addition to the nine senescence-related genes CMV-infection triggered the upregulation of additional 26 immunoregulatory genes in early passage fibroblasts (see Table 1). These were mainly genes regulating apoptosis and the NFkB pathway. The expression of all 84 investigated genes was measured at four different time points (6, 24, 48 and 72 hours) after CMV-infection. Kinetics of gene regulation were variable for the individual genes and revealed three patterns of gene expression as shown in Figure 2. In most cases genes were maximally upregulated 24 hours post infection and either returned to basal level after 48 hours (Figure 2A) or showed a relatively stable activation (Figure 2B). Only few genes showed a continuous upregulation with a maximum at 72 hours post infection (Figure 2C) and none of the regulated genes was maximally upregulated 6 or 48 hours post infection. The maximally observed fold regulation of the genes following CMV-infection is shown in Table 1. Superarray data were validated and confirmed using qRT-PCR for several exemplary genes (data not shown).

In summary these data show that CMV influences the mRNA expression of immunity-related host cell genes and thereby causes a modification of the cellular microenvironment. Furthermore we demonstrate that beyond partially imitating senescence-related inflammation CMV induces a robust inflammatory response in fibroblasts, which may contribute to age-related inflammation later in life. We cannot exclude that infection with other viruses might induce similar responses. However, CMV has particularly been linked with cellular senescence [8,9,15]. In this context IL6 is of particular interest since previous studies showed that high IL6 expression in combination with CMV-infection is associated with an increased risk of frailty in elderly people [16]. In general, virus-induced alterations in the cellular expression of cytokines such as IL6 may be important for the activation of specific leucocytes and their recruitment to the site of infection, thereby playing a role in viral pathogenesis. However, inappropriate production of immunostimulatory molecules leads to the excessive activation of the immune system and thereby to chronic inflammation. Since CMV causes life-long chronic latency in different human cells, leading to chronic antigenic stimulation and to increased production of immunity-related genes, CMV contributes to inflamm-aging and aggravates immunosenescence.

**Figure 3:**
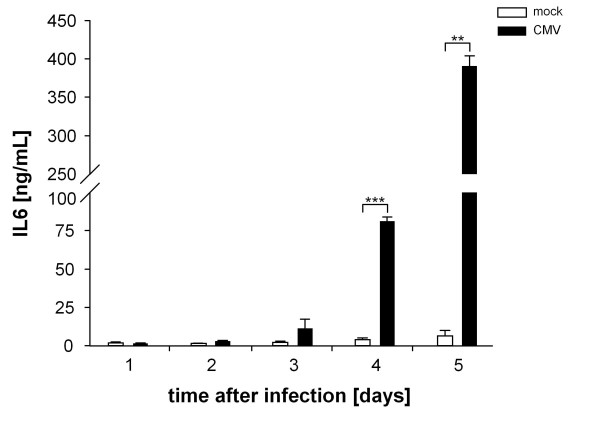
**IL6 protein levels in the supernatant of human fibroblasts following *in vitro *infection with CMV**. Human lung fibroblasts were left untreated (mock) or infected with the CMV strain Town-eGFP. IL6 protein secretion of mock- and CMV-infected fibroblasts was analyzed by ELISA over a time period of 5 days. The arithmetic mean ± S.E.M. were calculated for each examined group and time point. Comparisons between two groups were analyzed by Student's t-test, using SPSS version 19.0 (SPSS Inc., Chicago, Illinois, USA). p values below 0.05 were considered as statistically significant. * p ≤ 0.05, ** p ≤ 0.01, *** p ≤ 0.001.

In order to demonstrate that the induction of immunity-related genes following CMV-infection is also translated on the protein level ELISA experiments were performed. IL6 cytokine secretion was chosen as an example and was measured in the supernatants of untreated and CMV-infected fibroblasts over a culture period of five days. As shown in Figure 3 mock-infected fibroblasts produced low levels of IL6, which accumulate in the supernatant over time. Upon CMV-infection high IL6 concentrations could be observed starting at day 3 confirming the gene regulation observed on transcriptional level.

In conclusion our results demonstrate that CMV not only affects the T cell pool but also induces inflammatory processes in human fibroblasts. Therefore early life infection with CMV and episodes of reactivation throughout life might induce immunoregulatory changes supporting inflammatory processes and disease later in life.

## Materials and methods

### Cells

Human diploid fetal lung fibroblasts (Mrc-5) were cultivated in DMEM (Gibco Invitrogen Corporation, Paisley, Scotland) supplemented with 10% FCS (Sigma-Aldrich, Vienna, Austria), 100 Units/mL penicillin, 100 μg/mL streptomycin (Invitrogen, Lofer, Austria) and 2 mM L-glutamine (Sigma-Aldrich) at 37°C and 5% CO_2_. For the experiments early passage fibroblasts (#22 - #26), showing an exponential growth behavior, and late passage fibroblasts (#45 - #48), that had entered the state of terminally growth arrest, were used. To evaluate the state of senescence of the fibroblasts, activity of SA-ß-galactosidase at pH 5.8 [17] and gene expression of p15, p16 and p21 were measured. For gene expression analysis early and late passage fibroblasts were used, whereas CMV-infection was performed with early passage fibroblasts.

### Virus

Human cytomegalovirus (strain Town-eGFP) was propagated in Mrc-5 cells. Infectious virus particles in the virus stock were quantified by a standard plaque assay. Briefly, 500 μL of varying virus dilutions were added to confluent Mrc-5 cells in a 12-well plate. After two hours of incubation (37°C, 5% CO_2_) virus suspension was removed, cell monolayers were covered with 2 mL DMEM containing 1% methylcellulose (Sigma-Aldrich) and incubated for 10 days. Then plaques were counted under a fluorescence microscope. The viral concentration is expressed as plaque-forming units per mL (pfu/mL). All *in vitro *experiments with the virus were performed with a multiplicity of infection (MOI) of 1.

### CMV-infection of fibroblasts

For CMV-infection fibroblasts were seeded in 6-well plates and maintained in supplemented DMEM until they reached 100% confluency. Culture medium was removed and replaced by virus-containing medium. Untreated fibroblasts were used as a control (mock-infected). Supernatants were collected over several days as indicated and used for cytokine profiling by ELISA. Additionally, cells were harvested for isolation of total mRNA.

### Total RNA-isolation and qRT-PCR

Total cellular RNA was extracted from fibroblasts using the RNeasy Plus Mini Kit (Qiagen, Hilden, Germany). 0.25-0.5 μg of total RNA was used for cDNA synthesis, which was performed using the Reverse Transcription system A3500 with Oligo (dT)-primers (Promega, Madison, USA). cDNA sequences were amplified by qRT-PCR using the 'Light Cycler^® ^480 System' with the 2 × LightCycler 480 SYBR Green 480 master mix (Roche, Basel, Switzerland). Sequence-specific oligonucleotide primers were designed using Primer3 software [18] and synthesized by MWG Biotech (Ebersberg, Germany).

### RT^2 ^Profiler PCR Array

To determine the profile of genes associated with the human innate and adaptive immune response (for a complete list refer to http://www.sabiosciences.com/rt_pcr_product/HTML/PAHS-052A.html) of untreated and CMV-infected fibroblasts, a RT^2 ^Profiler PCR Array (SA Biosciences™/Qiagen) was used. The obtained data were analyzed using a Web-Based Data Analysis tool (http://pcrdataanalysis.sabiosciences.com/pcr/arrayanalysis.php).

### Cytokine profiling

Supernatants of fibroblasts were analyzed in duplicates for the presence of IL6 protein by a commercially available ELISA kit (MabTech, Hamburg, Germany).

## Competing interests

The authors declare that they have no competing interests.

## Authors' contributions

JW carried out the *in vitro *experiments with the virus, performed the superarray, ELISA and the qRT-PCR, analyzed the data and prepared the manuscript. BW prepared the CMV stock and participated in the design of the study, analysis of the data and preparation of the manuscript. BGL designed the study and supervised the preparation of the manuscript. All authors read and approved the final manuscript.
